# Compound 13 Promotes Epidermal Healing in Mouse Fetuses via Activation of AMPK

**DOI:** 10.3390/biomedicines11041013

**Published:** 2023-03-27

**Authors:** Kento Takaya, Keisuke Okabe, Shigeki Sakai, Noriko Aramaki-Hattori, Toru Asou, Kazuo Kishi

**Affiliations:** Department of Plastic and Reconstructive Surgery, Keio University School of Medicine, Tokyo 160-8582, Japan

**Keywords:** actin cable, AMPK, compound 13, epidermal healing, mouse fetus

## Abstract

Unlike adults, early developing fetuses can completely regenerate tissue, and replicating this could lead to the development of treatments to reduce scarring. Mice epidermal structures, including wound healing patterns, are regenerated until embryonic day (E) 13, leaving visible scars thereafter. These patterns require actin cable formation at the epithelial wound margin through AMP-activated protein kinase (AMPK) activation. We aimed to investigate whether the administration of compound 13 (C13), a recently discovered AMPK activator, to the wound could reproduce this actin remodeling and skin regeneration pattern through its AMPK activating effect. The C13 administration resulted in partial formations of actin cables, which would normally result in scarring, and scar reduction during the healing of full-layer skin defects that occurred in E14 and E15 fetuses. Furthermore, C13 was found to cause AMPK activation in these embryonic mouse epidermal cells. Along with AMPK activation, Rac1 signaling, which is involved in leaflet pseudopodia formation and cell migration, was suppressed in C13-treated wounds, indicating that C13 inhibits epidermal cell migration. This suggests that actin may be mobilized by C13 for cable formation. Administration of C13 to wounds may achieve wound healing similar to regenerative wound healing patterns and may be a potential candidate for new treatments to heal scars.

## 1. Introduction

Complete wound regeneration preserves skin structure and physiological function, including sensation and perception of stimuli, whereas incomplete wound regeneration results in fibrosis and scarring. Wounds in adult mammals heal without regeneration and are replaced with scar tissue [1]. Scar tissue is considered to be characterized by the following factors: (i) fibrosis of the dermis, (ii) loss of skin texture, (iii) loss of skin appendages, and (iv) change in color tone [2]. Therefore, “scarless wound healing” will only be achieved when the mechanism of regeneration in each parameter is clarified.

In order to elucidate the mechanism of wound regeneration, it is necessary to mimic the behavior of molecules in animals that have the ability to regenerate. *Danio rerio* and *Xenopus laevis* have long been the representatives of such animals, but the skin of amphibians and fish differs from that of mammals [3,4]. In mammals, complete scar regeneration occurs early in the mouse developmental stage, switching between periods of visible and histological scarring as development progresses [5]. In particular, it has been shown that, in the regeneration of the epidermis, including texture, there are switches during embryonic (E)13 and E14 days, and this regeneration involves the action of actin fibers at the wound margin [6]. This actin-cable type of embryonic wound healing is a conserved process in which peri-wound cells migrate to the wound area, promoting rapid and scarless repair. The concept of actin cables, which was first reported in chick embryos [7], occurs due to the polymerization of actomyosin at the limbus of a single epidermal cell at the wound margin and its contraction [8]. However, during the non-regenerative period of the epidermis, healing occurs through a pattern of epidermal cell migration over the exposed connective tissue [6,9]. Therefore, epidermal regeneration may be obtained through inhibiting the pattern of cell migration and inducing actin cable formation.

Recent experiments have proposed that factors controlling wound closure mechanisms, including actin cables, involve substrate adhesion, wound size, and shape [10,11,12,13]. The environment in which actin cables form is limited, and on adhesive substrates, the cell crawling mechanism is believed to predominate over wound closure, especially in large wounds. In such situations, the wound boundaries are usually roughened during the closure process [12]. Leader cells with a fingertip-like structure may also be observed at the anterior margin. On nonadherent substrates where cells cannot crawl up, purse-string-like contraction by actomyosin rings on top of the cells seems to be an important mechanism for wound closure. In this case, the wound edge is usually smooth and the wound shape is often rounded during this process [13]. Actomyosin cables behave differently depending on the shape of the wound, either by pulling concave edges forward and by pulling convex edges backward. The two mechanisms are not mutually exclusive; rather, they often coexist and influence each other [14]. However, the intrinsic complexity of the behavior of this cell population and its contribution to actin cable formation is unknown, and inducing actin cable formation in either environment leads to a recapitulation of embryonic type of wound healing.

This actin cable formation requires activation of AMP-activated protein kinase (AMPK) [6,15], which is involved in the regulation of microtubule and actin filament behavior [16]. Furthermore, AMPK and its substrate PDLIM5 inhibit cell migration through suppressing the Ras-related C3 botulinum toxin substrate 1 (Rac1)-actin-related protein (Arp) 2/3 signaling pathway [17].

The relationship between AMPK activation and scarring has been previously reported as being able to rescue MSC/fibroblast/myofibroblast defects, as well as defect phenotypes in vitro via the AMPK-dependent pathways [18]. It has also been reported that AICAR-dependent AMPK signaling leads to greater activation of endogenous CD44 pos CD45 neg fibroblast precursors, regulates differentiation into fibroblasts and maturation into myofibroblasts, and improves scar formation [19].

Recently, 5-(5-hydroxyl-isoxazol-3-yl)-furan-2-phosphonic acid (compound 2, C2) and its prodrug (compound 13, C13) were identified as potent allosteric activators of AMPK through the screening of a library of AMP mimics. They were shown to stimulate AMPK and inhibit hepatic lipogenesis in vivo [20], and C13, as a potent allosteric activator of the α1 complex, was shown to cause Thr172 dephosphorylation as well as AMP [21]. However, the effect of C13 on wound healing has not yet been addressed.

We hypothesized that the administration of C13 to acute wounds in fetal mouse embryos would alter actin dynamics via the activation of AMPK and promote skin regeneration or healing. Using our proprietary mouse fetal wound healing model, we administered C13 to fetal wounds at a time when skin was not regenerating and observed the effect on wound healing. Since fetal mouse wounds heal within 48–72 h, we focused on the 24 h and 48 h post-injury time points [6]. Our findings indicated that C13 may be a potential candidate for a new drug that induces epidermal regenerative wound healing.

## 2. Materials and Methods

### 2.1. Ethical Consideration

The research protocol was reviewed and approved by the Animal Experimentation Committee of Keio University School of Medicine (approval number: 20170914). All experiments were performed in accordance with institutional guidelines for animal experimentation at Keio University. This study is reported in accordance with the Animal Research: Reporting of In Vivo Experiments (ARRIVE) guidelines.

### 2.2. Fetal Wounding Procedure

Pregnant female ICR mice were used in this experiment. All mice were obtained from Sankyo Laboratory Services (Tokyo, Japan). During rearing, vaginal plugs were checked twice daily, and if a plug was observed, the fetus was designated E0 at that time. Fetuses were injured at E14 and E15. Surgeries were performed on four pregnant mice at each time point, creating wounds in at least four fetuses. The mothers were also divided into a control group and a C13-treated group, with 8 fetuses in each group. The surgery was performed under inhalation anesthesia with isoflurane, and the skin and abdominal wall were incised with surgical scissors to expose the uterus. Using an operating microscope, the myometrium, amnion, and yolk sac were incised with microsurgical scissors. A full-layer incision wound approximately 2 mm long was created in the fetal lateral thoracic region with the scissors. At the time of surgery to create the wound, 100 μL of 0.01% dimethyl sulfoxide (DMSO) was administered to the control group and 100 μL of C13 (1 nM, Selleck Chemicals, Houston, TX, USA) dissolved in 0.01% DMSO was administered to the C13 group in the amniotic fluid. After wounding, the fetus in E14, the amnion, and the yolk sac were sutured with 9-0 nylon, but the myometrium was left open without sutures to prevent uterine rupture. In contrast, at E15, after fetal wound creation, the myometrium was sutured with 9-0 nylon, the uterus was returned to the abdominal cavity, and the abdomen was closed. Ritodrine hydrochloride (Fujifilm Wako Pure Chemicals, Osaka, Japan), a uterine relaxant, was then administered intraperitoneally at 1 μg/g body weight just before wound closure. The peritoneum and skin were then continuously sutured with 5-0 nylon thread. The maternal mice were euthanized by cervical dislocation, and the fetuses were harvested 24, 48, and 72 h after injury. The wounds were examined using a VHX-8000 ultra-high precision digital microscope (Keyence, Osaka, Japan). Skin was harvested from the fetal wounds under a microscope and fixed in 4% paraformaldehyde (PFA) for 24 h. The fixed tissues were embedded in paraffin and stained. For immunostaining, the fixed tissues were soaked in 20% sucrose/PBS, frozen, embedded in OCT compound (Sakura Finetek Japan Co., Ltd., Tokyo, Japan), and sliced at 7 μm by using a mictotome.

### 2.3. Cell Assay

The skin tissues of mouse fetuses at E14 and E15 were immersed in dispase (Fujifilm Wako Chemical, 500 U/mL) and treated at 37 °C for 24 h to separate the epidermis from the dermis. The epidermis was cut and placed on a dish, and medium was added to obtain fetal mouse epidermal cells. The cells were plated in 96-well plates (5 × 10^3^ cells per well, n = 3) and maintained in 100 μL of medium. Thereafter, C13 was administered 24 h later to each well at a final concentration of 1 nM. After 24 h of incubation, the cells were collected, and RNA and protein were obtained as described below.

### 2.4. Immunohistochemistry

The mouse wound tissue was placed directly on glass slides and washed three times with 0.2% Triton X-100 in PBS (PBST) for 10 min. For blocking of nonspecific antigens, the tissue was incubated in 3% bovine serum albumin (BSA)/PBS solution for 1 h at room temperature (20–25 °C). The cells were then incubated overnight at 4 °C with Acti-stain 488 phalloidin (PHDG1-A; Cytoskeleton, Inc., Denver, CO, USA; 1:140) for actin staining; after washing three times with PBST, nuclei were labeled with Cellstain DAPI Solution (Fujifilm Wako Pure Chemicals; 1:500) and mounted on glass slides with ProLong Gold (Invitrogen, Waltham, MA, USA). All slides were observed with a confocal laser scanning microscope (FLUO-VIEW FV3000, Olympus, Tokyo, Japan).

### 2.5. Laser Microdissection (LMD), RNA Isolation, and Reverse Transcription

We performed LMD using a PALM MicroBeam (Carl Zeiss, Oberkochen, Germany) to collect RNA from the tissue. Briefly, the manufacturer’s recommended slides and collection tubes (AdhesiveCap 500 opaque, Carl Zeiss) were set up, and the samples were collected from the wound margins by carefully cutting the tissue while observing with a 20× magnification objective lens. The tube cap was filled with Buffer RLT (RNeasy microkit, Qiagen, Hilden, Germany) and filled with β-mercaptoethanol to allow the separation of intact RNA using the RNeasy microkit (Qiagen). Total RNA was extracted from the cells or skin tissues according to the manufacturer’s instructions, and then placed in a T100^TM^ thermal cycler (Bio-Rad, Hercules, CA, USA) along with Maxima™ H Minus cDNA Synthesis Master Mix (ThermoFisher Scientific, Waltham, MA, USA) at 25 °C for 5 min, 55 °C for 10 min, and 80 °C for 10 min to thermally inactivate revertase and produce cDNA.

### 2.6. Quantitative Real-Time PCR

RT-qPCR was performed using an Applied Biosystems 7500 Fast Real-Time PCR System (Thermo Fisher Scientific). A total of 40 cycles were performed, and the fluorescence of each sample was measured at the end of each cycle. The PCR reaction was performed in two major steps: holding the reagent at 95 °C for 3 s (denaturation) and at 60 °C for 30 s (annealing and extension). In the subsequent melting curve analysis phase, the temperature was increased from 60 °C to 95 °C and fluorescence was measured continuously. Rac1 (Mm01201653_mH), Arp2 (Mm07300461_g1), and Arp3 (Mm02342769_g1) were used as the primers (all from Thermo Fisher Scientific); PCR master mix (Cat. 4352042; Applied Biosystems, Foster City, CA, USA) was used according to the manufacturer’s instructions; and ACTB (Mm02619580_g1, ThermoFisher Scientific) was used as a control gene for normalization. The gene expression levels at normal sites were used as baseline, and fold-change values were determined using the 2^−ΔΔCt^ method.

### 2.7. Western Blotting

Total proteins were extracted from the cells and tissues with RIPA buffer (Santa Cruz Biotechnology, Santa Cruz, CA, USA). Each sample (40 μg) was electrophoresed on 10–20% polyacrylamide gels (Mini-PROTEAN^®^ TGX™ Precast Gels; Bio-Rad) and transferred to a Trans-Blot Turbo Transfer System (Bio-Rad). After blocking with 3% nonfat milk at room temperature for 1 h, the primary antibodies, AMPKα (1:100, Cell Signaling technology, Danvers, MA, USA), Phospho-AMPKα (Thr172) (1:100; Cell Signaling technology), and GAPDH (1:2000 dilution; Santa Cruz Biotechnology), diluted in a blocking solution were used to incubate the samples at 4 °C overnight. The next day, the samples were incubated with the following secondary antibodies at 37 °C for 1 h: donkey anti-goat IgG H&L (HRP) (ab6885; Abcam) and goat anti-rabbit IgG H&L (HRP) (ab205718; Abcam). After washing, immunoreactive protein bands were visualized using an ECL Western Blotting Substrate (Promega, Madison, WI, USA). Images of the bands were obtained using a chemiluminescence imager (ImageQuant LAS4000mini; GE Healthcare, Chicago, IL, USA). Image analysis was performed using ImageJ (version 1.53t, NIH, Bethesda, MD, USA). Each experiment was repeated three times.

### 2.8. Cell Migration Assay

The epidermal cells of E14 and E15 were treated with mitomycin C (Nakarai Tesque, Inc., Kyoto, Japan) (10 µg/mL, 37 °C for 3 h) to remove their proliferative effects before assay. The cells were grown to confluence on a plastic dish in Dulbecco’s modified Eagle’s medium at 37 °C, 5% CO_2_, and 95% relative humidity for up to 24 h. The surface of the cells was scratched 500 µm with a pipette tip. When the cells were grown, C13 was administered to each well at a final concentration of 1 nM. As a control, an equal volume of DMSO was added, and after 24 h of incubation, cell migration from the abraded edge was visualized under a microscope (BZ-X800; KEYENCE, Osaka, Japan).

### 2.9. Rac1 Activation Assay

The proteins extracted by the method described above were used―Rac1 activation was assessed with the G-LISA Rac1 Activation Assay Biochem Kit (Cytoskeleton inc., Denver, CO, USA) according to the manufacturer’s protocol. Briefly, 50 µL of protein adjusted to 0.5 mg/mL was added to a special 96-well plate of the kit and incubated in a cold orbital microplate shaker (Biosan, Riga, Latvia) for 30 min at 4 °C. An antigen-presenting buffer was added for 2 min, and then a 1:300 dilution of anti-Rac1 antibody in the kit was added, allowing the mixture to react at room temperature for 45 min. After washing, HRP-conjugated secondary antibody diluted at 1:100 was added and allowed to react at room temperature for 45 min. After washing, a HRP detection reagent was added and absorbance was detected at 490 nm with a microplate reader (SpectraMax Paradigm, Molecular Devices, San Jose, CA, USA). Each experiment was repeated three times.

### 2.10. Statistical Analysis

Statistically significant differences between the two groups were determined using the Statistica software version 9.0 (StatSoft, Tulsa, OK, USA). Differences were considered significant at *p* < 0.05. A Mann–Whitney U test was used to analyze the differences between the two groups.

## 3. Results

### 3.1. C13 Promotes Epidermal Healing at E14 and E15

**Figure 1:**
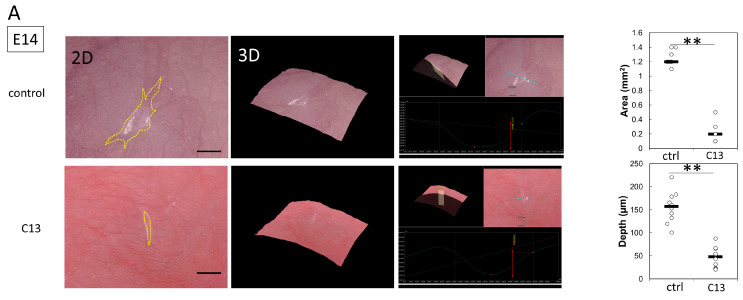

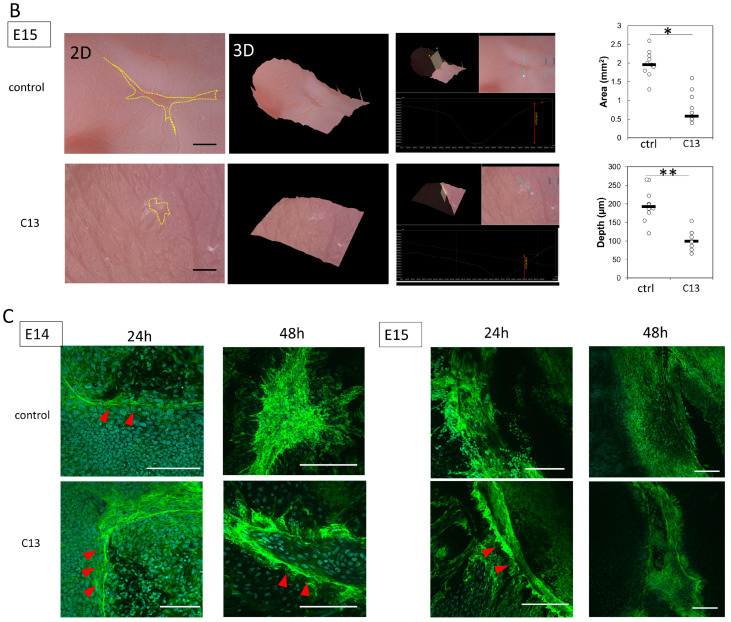
The effect of C13 administration on fetal mouse wound healing. (**A**) Wound evaluation of salicylic acid administration at E14 (bar, 500 µm), (**B**) wound evaluation of salicylic acid administration at E15, and (**C**) microscopic observation of actin cable formation during wound healing in E14 and E15 mouse fetuses. Green fluorescence is phalloidin, indicating actin filaments, and red arrows indicate areas of actin cable formation (bar, 20 µm). Ctrl, control; C13, compound 13. * *p* < 0.05, and ** *p* < 0.01.

We first investigated the effect of C13 administration on fetal mouse wound healing. Since the skin did not regenerate after E14, leaving a visible scar, we administered C13 and the control solution to the wounds at E14 and E15 and observed the wounds 72 h later. The results showed a significant reduction in visible scar size and depth at E14 (control vs. C13, area: *p* = 0.00017; depth: *p* = 0.00021) (Figure 1A).

Similarly, at E15, the scar size and depth were significantly reduced in the C13 group (control vs. C13, area: *p* = 0.011; depth: *p* = 0.0017) (Figure 1B). Thus, C13 administration reduced scarring.

We investigated whether actin cable formation is involved in this phenotype. Normally, actin cables are formed at E14 up to 24 h after injury, after which the healing process switches to a migratory cellular healing pattern. Furthermore, actin cables are not formed after E15. In the present experiment, at E14, cables were observed at 24 h in the controls, but a switch to cell migration occurred at 48 h, which was consistent with previous reports, whereas a partial actin cable-like structure was observed at the wound margin at 48 h after C13 administration (Figure 1C). At E15, no actin cable structure was observed at the wound margin during wound healing in the control group; however, a partial cable-like structure was observed at the wound margin at 24 h in the C13-treated group. Moreover, this cable structure disappeared 48 h later (Figure 1D). Thus, C13 induces actin cable formation during wound healing.

### 3.2. C13 Activates AMPK in Mouse Epidermal Cells and at Wound Edges in Embryos

**Figure 2:**
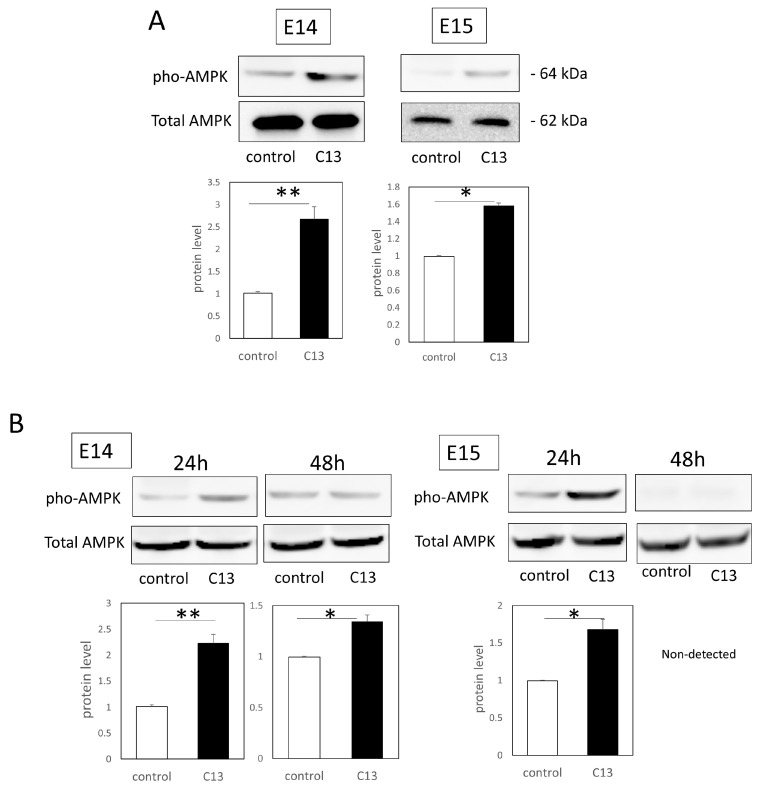
Alteration of AMPK activity with C13 in fetal mouse epidermis. (**A**)The effect of C13 on mouse embryonic epidermal keratinocytes at E14 and E15, and (**B**) the effect of C13 on the wound healing process of mouse fetuses at E14 and E15. The total AMPK expression level was used to determine the AMPK expression level of the phosphorylated form of AMPK. * *p* < 0.05, and ** *p* < 0.01.

We next investigated whether C13 elicited AMPK activity in the wound. First, the treatment of E14 and E15 mouse embryonic epidermal keratinocytes with C13 and the controls was compared. We found that expression of the phosphorylated form of AMPK was predominantly elevated after 24 h (E14, *p* = 0.000021; E15, *p* = 0.012) (Figure 2A).

Therefore, since C13 might activate AMPK in fetal mouse epidermis, we analyzed AMPK signaling proteins expressed at the wound margin in vivo. For E14 fetal mouse epidermis, the phosphorylated form of AMPK expression was significantly elevated at both 24 h and 48 h post-wounding (24 h, *p* = 0.00032; 48 h, *p* = 0.018). For E15 fetal mouse epidermis, the phosphorylated form of AMPK expression was also significantly elevated in the C13 group at 24 h after wounding (*p* = 0.00011), but no phosphorylated form of AMPK expression was observed at 24 h (Figure 2B).

### 3.3. C13 Suppresses Rac1 Signaling and Cell Migration in Mouse Epidermal Cells and at Fetal Wound Borders

**Figure 3:**
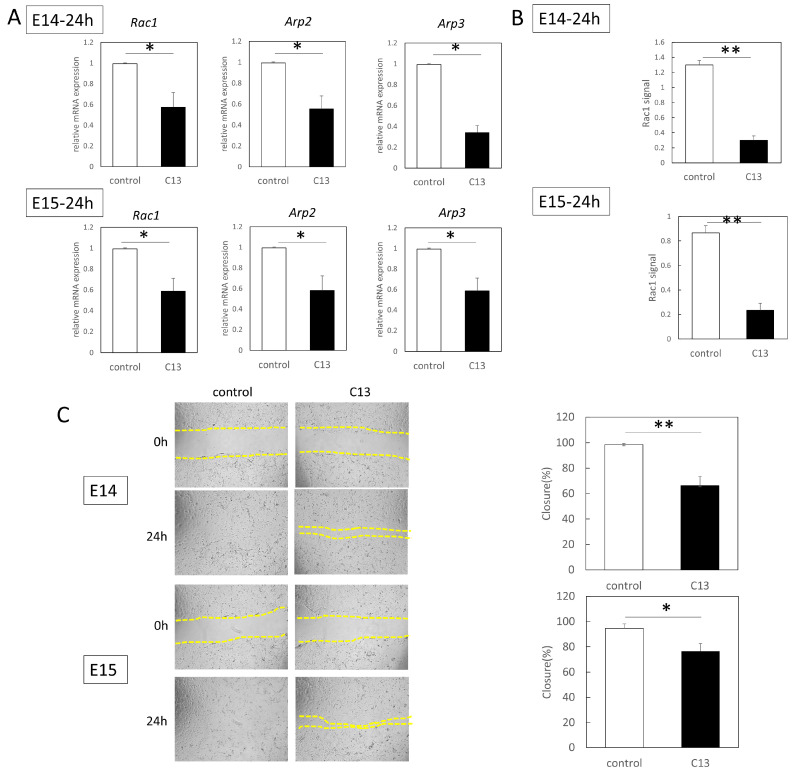
Suppression of Rac1 signaling and cell migration by C13 in fetal mouse epidermis. (**A**) Comparison of Rac1 signaling-related gene expression at 24 h post-injury. Gene expression levels were normalized using *ACTB* as an endogenous control. (**B**) Comparison of Rac1 signals at 24 h post-injury. Absorbance was read at 490 nm and the background was subtracted for comparison. (**C**) Migration assay of E14 and E15 mouse epidermal cells. The histogram on the right shows a comparison of the quantitative analysis of the scratch area. Migration is significantly inhibited in the C13 treatment group. * *p* < 0.05, and ** *p* < 0.01.

When the expression of Rac1, which is a molecule that reconstitutes actin into leaf pseudopodia formation, and its downstream molecules Arp2 and Arp3 were investigated, Rac1, Arp2, and Arp3 were all significantly downregulated at the gene level for E14 and E15 fetal mouse epidermis (Figure 2C). Rac1 activation assay was performed to evaluate the degree of Rac1 activity. In the migration assay, the migration capacity of both E14 and E15 epidermal cells was significantly decreased following C13 administration (E14, *p* = 0.0017; E15, *p* = 0.021) (Figure 3B).

## 4. Discussion

Epithelial wound healing is a fundamental physiological process, and wounds must close to reestablish epithelial integrity and tissue physiology in multicellular organisms. This process involves two major mechanisms: coordinated cell crawling through lamellipodial, and filopodial projections and contraction of purse strings by actomyosin rings on the cells surrounding the wound. This is a complex collective behavior. Actomyosin contractility is usually an important regulator in cell population movement. We hypothesized that epithelial regeneration is assisted by C13, an AMPK activator, based on previous reports that contraction by actin cable formation is involved in the mechanism of complete epithelial regeneration in mouse fetuses and that AMPK activity is involved in actin dynamics.

In this study, we hypothesized that actin would be mobilized for the formation of filopodia for cell migration during the developmental stage of mouse fetuses when wounds do not regenerate. At E14, when wounds are normally not healed yet, C13 administration partially induced actin cable formation and reduced the depth and size of the wounds.

At E15, when actin cables do not normally form, partial cables were observed at 24 h post-injury, and the wound was reduced in both depth and size.

Furthermore, since C13 treatment induced AMPK phosphorylation in mouse epidermal cells, we investigated AMPK kinetics during wound healing. We found that C13 treatment caused phosphorylation of AMPK in the wound, coinciding with the timing of actin cable formation, and suppressed Rac1 signaling, a molecule involved in the formation of filopodia.

While AMPK has been implicated in atherosclerosis and cancer metastasis, little is known concerning its underlying mechanisms [22]. However, AMPK’s ability to regulate cell migration suggests that it is associated with wound healing [23]. In particular, administration of drugs such as AICAR and A769662, which cause AMPK activation, has been reported to cause actin cytoskeleton remodeling in epithelial cells [11]. In our previous study, administration of AICAR reduced scarring in mouse fetuses [6]. The identified AMPK activators are primarily selective for β1 subunit-containing AMPK complexes, which offers an opportunity to study the role of various β subunit isoforms [24,25]. However, its usefulness is limited when studying AMPK function in cells or tissues that express primarily β2-containing complexes. Unlike these AMPK activators, C13 is completely selective for AMPK when compared with other AMP-regulated or metabolizing enzymes and has the detailed characterization and potential mechanism of action of an AMP-mimetic but α1-selective AMPK activator [26]. α1-containing complexes are preferred, which may limit their use for some indications; however, along with β-isoform selective activators, such as A769662 and AICAR, this is an indication that α-isoform specific activators can be developed. Elucidating the isoforms in epidermal cells involved in wound healing will provide a basis for determining more correct indications for these AMPK activators.

Another important finding of this study is that while the actin skeleton was remodeled via AMPK activation, Rac1, which is involved in leaflet pseudopodia formation, was suppressed and cell migration was inhibited. One recent study reported that the inhibition of vascular smooth muscle cell migration through the Pdlim5 pathway by metformin, another AMPK activator, inhibited the progression of atherosclerosis, which is accelerated in diabetes [27]. In other words, our results suggest that AMPK activation may inhibit Rac1 activation and mobilize actin cable formation instead of leaflet pseudopodia formation and cell migration in mouse epithelial cells.

Indeed, recent reports indicate that aggregating cell migration in mechanical heterogeneity is driven by Rac1, or ROCK and myosin IIA-dependent cytoskeletal tension, and that cytoskeletal tension across the epithelial tissue is linked to F-actin and myosin-IIA throughout [28]. In other words, they cooperate to form a skirting structure that modulates collective tissue behavior on an exogenous matrix with mechanical heterogeneity. Thus, it is suggested that Rac1 regulation via AMPK activity may also be involved in collective cell migration that initiates tissue growth, remodeling, and morphogenesis by directing cell contractility.

This study had some limitations. With reference to the available reports, actin cables form only in the epithelium, and the effects of C13 on regeneration and repair of dermal and fascial structures have not been fully examined. Thus, the involvement of fibroblasts or myofibroblasts and α-smooth muscle actin expression in wound contraction and scar formation is unknown. However, we found that actin polymerization was also observed in the dermis at the wound margins, which warrants further investigation. In this study, C13 might also have a positive effect on postnatal wounds as it promoted wound healing through causing actin cable formation at E15, a pattern similar to that in neonates and adult animals. The effects of C13 on adult animal wounds are currently under investigation. In addition, the structure of mouse and human skin is different, and further experiments are needed to determine whether the effects of C13 can be applied to humans, as well as the possibility of side effects.

In conclusion, C13 administration was shown to promote wound healing in mouse fetuses via AMPK activation. This finding may have potential for the development of new therapeutic modalities to regenerate wounds.

## Data Availability

The data presented in this study are available from the corresponding author upon request.
